# Supplementary material for: BMAL1 and ARNT enable circadian HIF2α responses in clear cell renal cell carcinoma
Source: Nat Commun. 2025 Jul 1;16:5834. doi: 10.1038/s41467-025-60904-0 (PMC12215836; doi:10.1038/s41467-025-60904-0)
Supplement: Supplementary file 2 — Reporting Summary [file 41467_2025_60904_MOESM2_ESM.pdf]

nature portfolio

Corresponding author(s): Katja A. Lamia

Last updated by author(s): May 12, 2025



# Reporting Summary

Nature Portfolio wishes to improve the reproducibility of the work that we publish. This form provides structure for consistency and transparency in reporting. For further information on Nature Portfolio policies, see our Editorial Policies and the Editorial Policy Checklist.

## Statistics

For all statistical analyses, confirm that the following items are present in the figure legend, table legend, main text, or Methods section.

| n/a | Confirmed | |
|---|---|---|
| ☐ | ☒ | The exact sample size (*n*) for each experimental group/condition, given as a discrete number and unit of measurement |
| ☐ | ☒ | A statement on whether measurements were taken from distinct samples or whether the same sample was measured repeatedly |
| ☐ | ☒ | The statistical test(s) used AND whether they are one- or two-sided<br>*Only common tests should be described solely by name; describe more complex techniques in the Methods section.* |
| ☐ | ☒ | A description of all covariates tested |
| ☐ | ☒ | A description of any assumptions or corrections, such as tests of normality and adjustment for multiple comparisons |
| ☐ | ☒ | A full description of the statistical parameters including central tendency (e.g. means) or other basic estimates (e.g. regression coefficient) AND variation (e.g. standard deviation) or associated estimates of uncertainty (e.g. confidence intervals) |
| ☐ | ☒ | For null hypothesis testing, the test statistic (e.g. *F*, *t*, *r*) with confidence intervals, effect sizes, degrees of freedom and *P* value noted<br>*Give P values as exact values whenever suitable.* |
| ☒ | ☐ | For Bayesian analysis, information on the choice of priors and Markov chain Monte Carlo settings |
| ☒ | ☐ | For hierarchical and complex designs, identification of the appropriate level for tests and full reporting of outcomes |
| ☒ | ☐ | Estimates of effect sizes (e.g. Cohen's *d*, Pearson's *r*), indicating how they were calculated |

*Our web collection on statistics for biologists contains articles on many of the points above.*

## Software and code

Policy information about availability of computer code

| | |
|---|---|
| Data collection | RNA sequencing data for five projects in The Cancer Genome Atlas (TCGA) and from the clinical proteomic tumor analysis consortium 3 (CPTAC3) were downloaded from the NIH genome data commons (https://portal.gdc.cancer.gov/). Dependency data (DepMap_Public_23Q4 +Score,_Chronos) for 37 kidney cell lines were downloaded from the Cancer Dependency Map portal (https://depmap.org/portal/) on February 29, 2024. |
| Data analysis | We used Rstudio and packages: rstatix, ggpubr, pheatmap, venneuler, and ggplot2, Fastqc 0.12.1 from the trim galore suite 0.6.10, Bowtie2 2.5.3, samtools 1.6 and sambamba 1.0, MACS2 2.2.9.1, bedtools 2.31.1 , HOMER 4.11, deeptools 3.5.5. We also used web-based softwares: Clock correlation distance analysis (https://hugheylab.shinyapps.io/deltaccd/) and WEB-based GEne SeT AnaLysis Toolkit -WebGestalt (https://www.webgestalt.org/) Expression of ARNT, ARNT2, BMAL1, and BMAL2 was extracted, analyzed, and visualized in Rstudio using packages rstatix and ggpubr. Clock correlation distance analysis was performed using the online tool available through the Hughey lab (https://hugheylab.shinyapps.io/deltaccd/). Statistical analysis and data visualization were performed in Rstudio using packages rstatix and ggpubr. All code used for data analysis and visualization is available at github.com/KatjaLamia/Mello_BMAL1_HIF2A |

For manuscripts utilizing custom algorithms or software that are central to the research but not yet described in published literature, software must be made available to editors and reviewers. We strongly encourage code deposition in a community repository (e.g. GitHub). See the Nature Portfolio guidelines for submitting code & software for further information.

## Data

Policy information about availability of data

All manuscripts must include a data availability statement. This statement should provide the following information, where applicable:

- Accession codes, unique identifiers, or web links for publicly available datasets
- A description of any restrictions on data availability
- For clinical datasets or third party data, please ensure that the statement adheres to our policy

RNA sequencing and CUT&RUN sequencing data were deposited into the Gene Expression Omnibus (GEO) database. The RNA-seq FASTQ files were deposited to the GEO repository (Accession GSE269336, GSE269339, GSE269340, GSE290779). The CUT&RUN-seq FASTQ files were deposited to the GEO repository (Accession GSE269334, GSE290669).

## Research involving human participants, their data, or biological material

Policy information about studies with human participants or human data. See also policy information about sex, gender (identity/presentation), and sexual orientation and race, ethnicity and racism.

| | |
|---|---|
| Reporting on sex and gender | We report results for experiments performed in male and female mice. Data from human subjects analyzed in the manuscript includes both males and females (combined). |
| Reporting on race, ethnicity, or other socially relevant groupings | not applicable |
| Population characteristics | not applicable |
| Recruitment | not applicable |
| Ethics oversight | not applicable |

Note that full information on the approval of the study protocol must also be provided in the manuscript.

# Field-specific reporting

Please select the one below that is the best fit for your research. If you are not sure, read the appropriate sections before making your selection.

[x] Life sciences  [ ] Behavioural & social sciences  [ ] Ecological, evolutionary & environmental sciences

For a reference copy of the document with all sections, see nature.com/documents/nr-reporting-summary-flat.pdf

# Life sciences study design

All studies must disclose on these points even when the disclosure is negative.

| | |
|---|---|
| Sample size | Analyses of publicly available data includes all available samples. Luciferase assays routinely use six samples per condition in each experiment based on observed variability in outcomes, and we report the mean of three independently replicated experiments. RNA sequencing and CUT&RUN ChIP sequencing experiments include three samples per condition to enable robust statistical analysis while containing costs. Cell growth assays (colony formation) include three or four samples per condition for each experiment and the results of one representative experiment among at least three replicates is shown. For xenograft measurements, ten hosts were initially used for each cell line to enable detection of a 20% or greater difference in tumor growth with 80% power with an alpha of 0.05 based on previously observed variability in tumor growth. Subsequent experiments used five hosts per condition because we found that the effect sizes were larger than expected so fewer measurements were needed to achieve the desired statistical power. |
| Data exclusions | One BMAL1 IP in shBMAL1 expressing 786O cells was discarded from CUT&RUN analysis because there were only 500,000 sequencing reads for this sample and all other samples had ~20M reads/sample. Nude mice harboring xenograft tumors that died before the experiment was completed (two mice treated with PT2399 at ZT12, one mouse treated with PT2399 at ZT0, two mice treated with vehicle at ZT12) were excluded from analysis. |
| Replication | Most cell-based experiments were successfully replicated at least three times. HIF2a and ARNT Western blots in synchronized 786O cells are from one independent experiment with duplicate samples. The BMAL1 Western blot in the same experiment matches many similar experimental replicates. Variability in outcomes is reflected in results where appropriate. Xenograft experiments were successfully replicated twice. |
| Randomization | N/A |
| Blinding | Investigators were blinded to sample identities where possible. Blind data collection for weekly murine xenograft measurements were performed by pulling cages at random for measuring and not identifying mouse identity until after measurements were collected. |

# Reporting for specific materials, systems and methods

We require information from authors about some types of materials, experimental systems and methods used in many studies. Here, indicate whether each material, system or method listed is relevant to your study. If you are not sure if a list item applies to your research, read the appropriate section before selecting a response.

## Materials & experimental systems

| n/a | Involved in the study |
| --- | --- |
| ☐ | ☒ Antibodies |
| ☐ | ☒ Eukaryotic cell lines |
| ☒ | ☐ Palaeontology and archaeology |
| ☐ | ☒ Animals and other organisms |
| ☒ | ☐ Clinical data |
| ☒ | ☐ Dual use research of concern |
| ☒ | ☐ Plants |

## Methods

| n/a | Involved in the study |
| --- | --- |
| ☐ | ☒ ChIP-seq |
| ☒ | ☐ Flow cytometry |
| ☒ | ☐ MRI-based neuroimaging |

## Antibodies

| | |
| --- | --- |
| Antibodies used | Primary antibodies used for Western blotting were anti-HA polyclonal (Sigma #H6908, Lot 0000259082), anti-Flag polyclonal (Sigma #F7425), anti-βActin (Sigma #A1978), anti-HIF2α polyclonal (Novus Biologicals #NB100-122, Lot CP), anti-BMAL1 polyclonal (Abcam #ab93806, Lot GR1559049, anti-BMAL1 monoclonal (VWR #102231-824), and anti-KU80 (Takara # Y40400, Lot A0100015). Secondary antibodies used were Goat Anti-Mouse IgG (H + L)-HRP Conjugate (Bio-Rad #1706516), Goat Anti-Rabbit IgG (H + L)-HRP Conjugate (Bio-Rad #1706515), Goat Anti-Guinea Pig IgG-HRP Conjugate (Sigma #A7289). The primary antibodies used for immunoprecipitation were 5 ug of rabbit mAb IgG isotype as a negative control (CST #66362, Lot 3), 2 ug of rabbit mAb tri-methyl-lys-4 (CST # C42D8, Lot 15), 1 ug of HIF-2α rabbit mAb (CST #59973, Lot 1), or 2 ug of BMAL1 rabbit mAb (CST #14020, Lot 4). |
| Validation | anti-HIF2a polyclonal (Novus Biologicals #NB100-122) is validated in PMID 26861754.<br>anti-BMAL1 polyclonal (Abcam #ab93806) is validated in Figure 4A.<br>anti-BMAL1 monoclonal (VWR #102231-824) validation is available from the manufacturer at https://digitalassets.avantorsciences.com/adaptivemedia/rendition?id=7e1bd7ce6054e6f0872f30bd3e5c96591a7984c3&vid=7e1bd7ce6054e6f0872f30bd3e5c96591a7984c3&prid=original&clid=SAPDAM.<br>anti-KU80 (Takara #Y40400) has been extensively validated as described by the manufacturer at https://www.takarabio.com/documents/User%20Manual/Y40400/Y40400_DS.pdf?srsltid=AfmBOoroFiRZas6Hl90YkoAOG_KcSD5ws-uPadjf7euHe7inVvzOq1eH<br>anti-tri-methyl-lys-3 (CST #C42D8) is validated by the manufacturer: https://www.cellsignal.com/products/primary-antibodies/tri-methyl-histone-h3-lys4-c42d8-rabbit-mab/9751?srsltid=AfmBOoqrZWssT-jMFMHAF-88xkQuPM_ul7P_6d9tOmsVx-JtF_BB-d6D<br>HIF-2a rabbit mAb is validated by the manufacturer:<br>https://www.cellsignal.com/products/primary-antibodies/hif-2a-d6t8v-rabbit-mab/59973?srsltid=AfmBOorL3om5EVsEzn6joUtYbUXbTH3we9fmylJb7shb-0nIhS3IoyXf<br>BMAL1 rabbit mAb (CST #14020) is validated in Figure 5D and by the manufacturer:<br>https://www.cellsignal.com/products/primary-antibodies/bmal1-d2l7g-rabbit-mab/14020 |

## Eukaryotic cell lines

Policy information about cell lines and Sex and Gender in Research

| | |
| --- | --- |
| Cell line source(s) | All cell lines were purchased from ATCC |
| Authentication | None of the cell lines used were independently authenticated. |
| Mycoplasma contamination | The cells were not tested for mycoplasma contamination. |
| Commonly misidentified lines (See ICLAC register) | None of the cell lines used are included in the ICLAC register of commonly misidentified cell lines. |

## Animals and other research organisms

Policy information about studies involving animals; ARRIVE guidelines recommended for reporting animal research, and Sex and Gender in Research

| | |
| --- | --- |
| Laboratory animals | Eight weeks old NIH-III Nude mice were used as hosts for xenograft tumors. They were housed in conditions with twelve hours of light and twelve hours of dark daily with humidity recorded between 19%-69% and temperature was 64.0-74.5 F. |
| Wild animals | N/A |

| | |
|---|---|
| Reporting on sex | Male and female mice were used as hosts and results are reported separately for each sex. Some experiments used only female mice as hosts because no sex-specific differences were observed. |
| Field-collected samples | N/A |
| Ethics oversight | All murine husbandry and experiments were in regulation with the Institutional Animal Care and Use Committee at the Scripps Research Institute (La Jolla, California) under protocol #10-0019. |

Note that full information on the approval of the study protocol must also be provided in the manuscript.

# Plants

| | |
|---|---|
| Seed stocks | N/A |
| Novel plant genotypes | N/A |
| Authentication | N/A |

# ChIP-seq

## Data deposition

☒ Confirm that both raw and final processed data have been deposited in a public database such as GEO.

☒ Confirm that you have deposited or provided access to graph files (e.g. BED files) for the called peaks.

| | |
|---|---|
| Data access links<br>*May remain private before publication.* | https://www.ncbi.nlm.nih.gov/geo/query/acc.cgi?acc=GSE269334<br>https://www.ncbi.nlm.nih.gov/geo/query/acc.cgi?acc=GSE290669 |
| Files in database submission | Raw paired-end sequencing files (fq.gz), bigWig (.bw), peak file (.bed) (except for negative control) |
| Genome browser session<br>(e.g. UCSC) | https://genome-euro.ucsc.edu/s/rmello%40scripps.edu/Mello_2024 |

## Methodology

| | |
|---|---|
| Replicates | BMAL1 and HIF2a IPs were performed in technical triplicate in both 786O cells and A498 cells expressing shControl or shBMAL1. IgG IPs were performed in technical duplicate in both 786O cells and A498 cells expressing shControl or shBMAL1. One BMAL1 IP in shBMAL1 expressing 786O cells was discarded from analysis because there were only 500,000 sequencing reads for this sample and all other samples had ~20M reads/sample. |
| Sequencing depth | Paired end reads 20 million reads/sample. |
| Antibodies | The primary antibodies used for immunoprecipitation were 5 ug of rabbit mAb IgG isotype as a negative control (CST #66362), 2 ug of rabbit mAb tri-methyl-lys-4 (CST # C42D8), 1 ug of HIF-2α rabbit mAb (CST #59973), or 2 ug of BMAL1 rabbit mAb (CST #14020). |
| Peak calling parameters | Peak calling was performed using MACS2 2.2.9.1, specifying parameters --keep-dup all --max-gap 400 –p 1e-5 |
| Data quality | peak calling used a stringent p-value threshold of 1e-5. |
| Software | reads underwent alignment to both the human genome hg19 and the yeast genome sacCer3 using Bowtie2 2.5.3, with the following parameters: --local --very-sensitive --fr --dovetail --no-mixed -I 10 -X 700<br><br>Alignment files (SAM) were then converted to BAM format, and subjected to filtering, and duplicate reads were removed using samtools 1.6 and sambamba 1.0<br><br>Peak calling was performed using MACS2 2.2.9.1, specifying parameters --keep-dup all --max-gap 400 –p 1e-5<br><br>Post-peak calling, filtering against the hg19 blacklist was executed using bedtools 2.31.1 with the intersect option<br><br>annotation and motif analysis of the peaks was carried out using HOMER 4.11, using annotatePeaks.pl and findMotifsGenome.pl options with the human genome hg19 reference<br><br>Peak functional annotation was directly done by Homer using -go option, or with WEB-based GEne SeT AnaLysis Toolkit -WebGestalt (https://www.webgestalt.org/) to identify gene ontologies and KEGG-related pathways after crossing peaks annotation with RNA-seq |

data.

Spike-in normalization with the aligned reads was achieved against the yeast genome sacCer3 with deeptools 3.5.5 using bamCoverage –scaleFactor --smoothLength 60 --extendReads 150 –centerReads to produce BigWig files. Spike-in scale factor values were calculated as described in the manufacturer protocol (CST #86652).

Profiles and heatmap were obtained by using computeMatrix –referencePoint center after spike-in normalization.

BigWig files were uploaded to the UCSC genome browser (https://genome-euro.ucsc.edu/index.html) and tracks were visualized against the human genome hg19
