# Supplementary material for: BMAL1 and ARNT enable circadian HIF2α responses in clear cell renal cell carcinoma
Source: Nat Commun. 2025 Jul 1;16:5834. doi: 10.1038/s41467-025-60904-0 (PMC12215836; doi:10.1038/s41467-025-60904-0)
Supplement: Supplementary file 1 — Supplementary Information [file 41467_2025_60904_MOESM1_ESM.docx]

BMAL1 and ARNT enable circadian HIF2α responses in clear cell renal cell carcinoma

Supplementary Information

Rebecca M. Mello^1,2^, Diego Gomez Ceballos^1,2^, Colby R. Sandate^3^, Sicong Wang^1,2^, Celine Jouffe^4,5^, Daniel Agudelo^4^, Nina Henriette Uhlenhaut^4,6^, Nicolas H. Thomä^3^, M. Celeste Simon^7,8^, Katja A. Lamia^1,2*^

^1^Department of Molecular and Cellular Biology, Scripps Research Institute, La Jolla, CA, 92037, USA.

^2^Department of Molecular Medicine, Scripps Research Institute, La Jolla, CA, 92037, USA.

^3^Friedrich Miescher Institute for Biomedical Research, Basel, Switzerland.

^4^Institute for Diabetes and Endocrinology (IDE), Helmholtz Munich, Ingolstaedter Landstr. 1, 85764, Neuherberg, Germany.

^5^Institute for Diabetes and Cancer (IDC), Helmholtz Munich, Ingolstaedter Landstr. 1, 85764, Neuherberg, Germany.

^6^Metabolic Programming, TUM School of Life Sciences & ZIEL Institute for Food and Health, Gregor-Mendel-Str. 2, 85354, Freising, Germany.

^7^Abramson Family Cancer Research Institute, Perelman School of Medicine; Philadelphia, Pennsylvania 19104, USA.

^8^Department of Cell and Developmental Biology, University of Pennsylvania, Philadelphia, Pennsylvania 19104, USA.

*Corresponding author. Email: [klamia@scripps.edu](mailto:klamia@scripps.edu)

**Figure S1. BMAL1 mRNA is elevated in ccRCC.** (**A,B**) Detection of *ARNT*, *ARNT2*, *BMAL1,* and *BMAL2* (transcripts per million, TPM) calculated from RNA sequencing data from ccRCC tumors and normal kidney samples (*A*) or tumors and adjacent normal tissues from the same patients (*B*) in clinical proteomic tumor analysis consortium (CPTAC-3) and cancer genome atlas projects: colorectal adenocarcinoma (COAD, n=82 tumor, n=82 adjacent non-tumor), lung adenocarcinoma (LUAD, n=59 tumor, n=59 adjacent non-tumor), breast cancer (BRCA, n=113 tumor, n=113 adjacent non-tumor), kidney clear cell renal cell carcinoma (KIRC, n=72 tumor, n=72 adjacent non-tumor), and renal papillary cancinoma (KIRP, n=100 tumor, n=100 adjacent non-tumor). * Padj < 0.05, ** Padj < 0.01, **** Padj < 0.0001 by two-way ANOVA with Tukey’s posthoc correction. In all boxplots, the box depicts the median and interquartile range (IQR), whiskers extend either to the minimum or maximum data point or 1.5*IQR beyond the box, whichever is shorter. Outliers (values beyond the whisker) are shown as dots. (**C**) Images of normal kidney and ccRCC human tissue sections stained with anti-BMAL1 antibody and counterstained with hematoxylin. Scale bar, 20 micrometers. Source data are provided as a Source Data file.

**Figure S2. Circadian rhythms are maintained in ccRCC in contrast to other tumor types.** (**A**) Clock correlation distance (CCD) heatmaps calculated from RNA sequencing data from normal human tissues (GTEX, reference) or from tumors and non-tumor samples from the same tissues in cancer genome atlas (TCGA) projects: colorectal adenocarcinoma (COAD), lung adenocarcinoma (LUAD), small cell lung cancer (LUSC), breast cancer (BRCA), kidney clear cell renal cell carcinoma (KIRC), and renal papillary cancinoma (KIRP). The table shows the number of samples in each group (N), calculated CCD values for non-tumor (CCD-N) or tumor (CCD-T) samples, and adjusted p values (pval). (**B**) Luminescence detected in 786O or RCC4 cells expressing VHL (green) or control (black) in conjunction with destabilized luciferase under the control of the PER2 promoter. Cellular circadian rhythms were synchronized by treatment with dexamethasone. Data for 786O cells represent one of two experiments with similar outcomes. The experiment shown included three biological replicates per group; the second experiment (not shown) included five biological replicates per group. Data for RCC4 cells represent a single experiment including five biological replicates per group. Source data are provided as a Source Data file.

**Figure S3. In RCC cell lines, HIF2A (a.k.a. EPAS1) dependency is correlated with dependencies for *ARNT* and *BMAL1*.** Correlation of dependency (CHRONOS) scores for *ARNT*, *ARNT2*, *BMAL1*, and *BMAL2* with those for *EPAS1* in RCC cell lines from DepMap ^1,2^. Source data are provided as a Source Data file.

**Figure S4. Depletion of BMAL1 alters many of the same genes that are affected by rescuing VHL function in 786O cells.** (**A,B**) Heatmaps depicting all differentially expressed genes (DEGs) (*A*), or DEGs in the Hallmark HYPOXIA gene set (*B*) in786O cells and WT8 cells expressing the indicated shRNAs. DEGs were identified using DESeq2 with a false discovery rate (FDR) cutoff of 0.1. (Note: WT8 cells are 786O cells engineered to express WT VHL). Source data are provided as a Source Data file.

**Figure S5. Characterization of ARNT- and BMAL1-dependent transcripts in 786O cells.** Distribution of coding sequence length, transcript length, genome span, 5’UTR length, 3’UTR length, and GC content for transcripts that exhibit reduced expression exclusively in 786O cells expressing *shARNT*, exclusively in 786O cells expressing *shBMAL1*, or in both compared to 786O cells expressing *shControl*. Each “List” of DEGs so defined (depicted in blue) is compared to the “background” of all transcripts detected in RNA prepared from 786O cells expressing *shControl* (depicted in red). Analysis performed using ShinyGO ^3^. Source data are provided as a Source Data file.

**Figure S6. Endogenous BMAL1 contributes to HIF2α target gene expression in A498 cells.** (**A**) Detection of HIF2α, ARNT, BMAL1, and ACTIN by immunoblot in A498 cells expressing the indicated shRNAs. The samples derive from the same experiment but one gel for BMAL1, HIF2α, and ACTIN, and another for ARNT and ACTIN were processed in parallel. Data from one of two independent experiments with similar results. (**B-E**) Venn diagrams (*B,C*) and heatmaps (*D,E*) depicting all differentially expressed genes (DEGs) (*B*), significantly downregulated genes (*C,D*) or downregulated genes in the Hallmark HYPOXIA gene set (*E*) in A498 cells expressing the indicated shRNAs. DEGs were identified using DESeq2 with a false discovery rate (FDR) cutoff of 0.1. (**F**) Boxplot depicting changes in gene expression in patient-derived xenografts (PDXs) treated with PT2399 (data from ^4^ including sensitive PDXs only) for genes grouped by whether their expression in A498 cells is decreased by *shARNT* and not by *shBMAL1* (ARNTsp, yellow), by *shBMAL1* and not by *shARNT* (BMAL1sp, purple), by either *shARNT* or *shBMAL1* (Overlap, salmon), or neither (NA, gray). **** P < 0.0001 by two-way ANOVA with Tukey’s correction. Boxes depict the median and interquartile range (IQR), whiskers extend either to the minimum or maximum data point or 1.5*IQR beyond the box, whichever is shorter. Outliers (values beyond the whisker) are shown as dots. (**G-I**) Volcano plots depicting the expression changes for individual genes in the groups depicted in (*F*). Genes with padj < 0.05 are colored in red (fold change > 1.5) or blue (fold change < 0.67) . (**J,K**) GOBP (*J*) or KEGG (*K*) pathways enriched among ARNT-specific or BMAL1-specific target genes in A498 cells. (**L,M**) Enrichment plots showing the impact of *shARNT* (*F*) or *shBMAL1* (*G*) on genes in the Hallmark HYPOXIA gene set. Source data are provided as a Source Data file.

**Figure S7. Characterization of ARNT- and BMAL1-dependent transcripts in A498 cells.** Distribution of coding sequence length, transcript length, genome span, 5’UTR length, 3’UTR length, and GC content for transcripts that exhibit reduced expression exclusively in A498 cells expressing *shARNT*, exclusively in A498 cells expressing *shBMAL1*, or in both compared to A498 cells expressing *shControl*. Each “List” of DEGs so defined (depicted in blue) is compared to the “background” of all transcripts detected in RNA prepared from A498 cells expressing *shControl* (depicted in red). Analysis performed using ShinyGO^3^. Source data are provided as a Source Data file.

**Figure S8. Impact of *shBMAL1* on HIF2α chromatin interactions.** (**A**) Pie chart distribution of HOMER peak annotation for BMAL1 and HIF2α peaks in 786O cells expressing *shControl*. (**B**) Venn diagram depicting the numbers of genomic sites identified in CUT&RUN samples prepared using BMAL1-specific antibodies from 786O cells expressing *shControl* or *shBMAL1.* (**C**) Left: Venn diagram depicting numbers of genomic sites identified in CUT&RUN samples prepared using HIF2α-specific antibodies from 786O cells expressing *shControl* or *shBMAL1*. Right: Top motifs enriched in chromatin associated with HIF2α in 786O cells expressing *shControl,* *shBMAL1*, or both (common) (**D**) Representative genome browser tracks for peaks located in *CDH6, BCL3,* and *SLC29A4* loci that were among the 393 that were detected in HIF2α CUT&RUN samples in cells expressing *shBMAL1* and were not detected in HIF2α CUT&RUN samples in 786O cells expressing *shControl*. Source data are provided as a Source Data file.

**Figure S9. BMAL1 influences recruitment of HIF2α to a subset of target genes**. (**A**) Venn diagram depicting the numbers of genomic sites (“peaks”) identified in chromatin fragments isolated by CUT&RUN procedure from A498 cells using antibodies recognizing BMAL1 (blue) or HIF2α (red). **(B**) Chromatin binding profiles of BMAL1 and HIF2α in CUT&RUN samples (n=3 per condition) prepared from A498 cells expressing the indicated shRNAs. Peaks are depicted in four groups: BMAL1 peaks in A498 cells expressing *shControl* (top row: 899 peaks), HIF2α peaks in A498 cells expressing *shControl* (second row: 894 peaks), peaks associated with both BMAL1 and HIF2α in A498 cells expressing *shControl* (third row: 83 peaks), or HIF2α peaks identified only in A498 cells expressing *shBMAL1* (bottom row: 1,464 peaks). (**C**) Transcription factor binding motifs enriched in chromatin associated with BMAL1, HIF2α, or both (common) in *shControl* cells. (**D**) Representative genome browser tracks for BMAL1 and HIF2α CUT&RUN in A498 cells expressing *shControl* or *shBMAL1*, showing peaks in *VEGFA*, *SERPINE1*, and *NR1D1* loci. Data represent merged read counts for triplicate samples for each condition. Source data are provided as a Source Data file.

**Figure S10. BMAL1-HIF2α heterodimers are sensitive to disruption by PT2399.** (**A**) Three dimensional structure (PDB ID: 7W80) containing the bHLH and PAS domains of ARNT (magenta) and HIF2α (beige) bound to PT2399. Ligand binding to the PAS-B domain of HIF2α leads to structural reorganization of HIF2α loop (shown in blue), which thereby clashes with a loop in ARNT (shown in green). (**B**) Detection of HIF2α, the indicated FLAG-tagged proteins, and ACTIN by immunoblot in lysates (input) or samples purified by immunoprecipitation of the FLAG tag from HEK293 cells treated with the indicated concentratios of PT2399. These two and Figure 7A show three experimental replicates that were quantitated to generate the data shown in Figure 7B (which includes only the quantitation of samples in which ARNT-FLAG or BMAL1-FLAG were transfected). (**C**) Volcano plot depicting differentially expressed genes in patient-derived xenografts that were sensitive or resistant to growth suppression by PT2399 in ^4^. Genes with padj < 0.05 are colored in red (FC > 1.5) or blue (FC < 0.67). (**D,E**) Detection of *ARNT*, *CLOCK*, *NR1D2*, and *CRY2* transcripts in patient-derived xenograft tumors collected from mice treated with PT2399 (red) or vehicle control (black) grouped by whether tumor growth was reduced by PT2399 (sensitive) or not (resistant). Data represent transcripts per million detected by sequencing RNA collected from 11 resistant or 12 sensitive PDX tumors. Source data are provided as a Source Data file.

**Figure S11 BMAL1-HIF2α heterodimers promote 786O xenograft tumor growth.** (**A**) BMAL1-CLOCK (PDB ID: 4F3L) and ARNT-HIF2α (PDB ID: 7W80) interfaces with conserved amino acids that contact HIF2α but not CLOCK highlighted in cyan or yellow. (**B**) Sequence alignment of ARNT and BMAL1 in the region depicted in (*A*). (**C,D**) Detection of HIF2α, FLAG-BMAL1, CLOCK, and ACTIN by immunoblot in lysates (input) or samples prepared by immunoprecipitation of the FLAG tag from HEK293 cells (*C*) or 786O cells (*D*) transiently expressing the indicated plasmids. Data represent a single experiment each. (**E,F**) Volume of xenograft tumors grown from 786O cells implanted in flanks of female NIH-III Nude mice treated with 10 mg/kg of PT2399 or vehicle control three times weekly by oral gavage between ZT0 and ZT2. Dark and light green symbols represent xenografts grown from 786O cells expressing wildtype (dark green) or D144A mutant (light green) BMAL1; filled and open symbols represent tumors in mice treated with vehicle control or with PT2399, respectively. n=5 animals per group. Panel (*E)* includes all data presented in Figure 8A-D on one graph. Panel (*F*) includes the data for all animals that were treated with PT2399. Source data are provided as a Source Data file.

**Figure S12 Circadian rhythms in 786O xenograft tumors.** (**A**) Detection of HIF2α and human-specific Ku80 by immunoblot in 786O cell xenograft tumors dissected from NIH-III Nude mice at the indicated zeitgeber times. Data from one of four independent experiments with similar results. The samples shown derive from the same experiment and were run on one gel, but the membrane was cut in half and one side was processed for HIF2α and the other half was processed for Ku80 in parallel. (**B**) Quantitation of Western blots like that shown in (*A*). Data represent the mean ± s.d. for four biological replicates. (**C**) Relative expression of the indicated transcripts measured by quantitative RT-PCR in four samles per ZT each measured in triplicate. Data represent the mean ± s.d. for four biological replicates each measured in triplicate. Data in (*B,C*) show quantitation of aggregated samples from four independent experiments. Source data are provided as a Source Data file.

**Figure S13 Tumor growth suppression respose to PT2399 treatment at ZT0 or ZT12.** Volume of xenograft tumors grown from 786O cells implanted in flanks of female NIH-III Nude mice treated with 10 mg/kg of PT2399 or vehicle control three times weekly by oral gavage at ZT0 (top graphs, open symbols) or ZT12 (bottom graphs, filled symbols) in two independently performed experiments that were initiated with n=5 animals per group. Four animals that died before the end of the experiment were excluded. Black and red symbols represent xenografts grown in mice treated with vehicle control or with PT2399 respectively. These plots show the volume measurements for tumors in individual mice that underlie the data shown in Figure 8F. Source data are provided as a Source Data file.

**Figure S14 Transcriptional response to PT2399 treatment at ZT0 or ZT12.** (**A,D,E**) Heatmaps depicting all differentially expressed genes (DEGs) (*A*) or DEGs in the Hallmark GLYCOLYSIS (*D*) or HYPOXIA (*E*) gene sets in xenograft tumors collected from mice six hours after they were treated with vehicle or PT2399 at ZT0 or at ZT12. DEGs were identified using DESeq2 with a false discovery rate (FDR) cutoff of 0.1. (**B**) Boxplot depicting changes in gene expression for transcripts that were increased (UP) or decreased (DOWN) by PT2399. Boxes depict the median and interquartile range (IQR), whiskers extend either to the minimum or maximum data point or 1.5*IQR beyond the box, whichever is shorter. **** Padj < 0.0001 by two-way ANOVA with Tukey’s posthoc correction. (**C, F**) Detection of the indicated transcripts in xenograft tumors collected from mice treated with PT2399 (red) or vehicle control (black) at ZT0 or at ZT12. * P < 0.05, ** P < 0.01, *** P < 0.001, **** P < 0.0001 by two-way ANOVA with Sidak’s correction for multiple hypothesis testing. Data represent three, four, or five biological replicates as indicated by the individual symbols, each measured in triplicate. Horizontal line depicts the mean. Five xenografts were established per group; one animal treated with vehicle at ZT12 and two animals treated with PT2399 at ZT12 were excluded because they died before the end of the experiment. Source data are provided as a Source Data file.

**Supplementary References**

1	Meyers, R. M. *et al.* Computational correction of copy number effect improves specificity of CRISPR-Cas9 essentiality screens in cancer cells. *Nat Genet* **49**, 1779-1784 (2017). <https://doi.org/10.1038/ng.3984>

2	Tsherniak, A. *et al.* Defining a Cancer Dependency Map. *Cell* **170**, 564-576 e516 (2017). <https://doi.org/10.1016/j.cell.2017.06.010>

3	Ge, S. X., Jung, D. & Yao, R. ShinyGO: a graphical gene-set enrichment tool for animals and plants. *Bioinformatics* **36**, 2628-2629 (2020). <https://doi.org/10.1093/bioinformatics/btz931>

4	Chen, W. *et al.* Targeting renal cell carcinoma with a HIF-2 antagonist. *Nature* **539**, 112-117 (2016). <https://doi.org/10.1038/nature19796>
